# Anti-Alopecia Activity of Coumarin Derivatives Isolated from *Merremia peltata* Leaves and Computational Study of Their Binding to Androgen Receptors Using Molecular Docking and Molecular Dynamic Simulation

**DOI:** 10.3390/ph16050669

**Published:** 2023-04-28

**Authors:** Syawal Abdurrahman, Ruslin Ruslin, Aliya Nur Hasanah, Mus Ifaya, Resmi Mustarichie

**Affiliations:** 1 Department of Pharmaceutical Analysis and Medicinal Chemistry, Faculty of Pharmacy, Universitas Padjadjaran, Sumedang 45363, Indonesia; 2 Department of Medical Laboratory Technology, Universitas Mandala Waluya, Kendari 93231, Indonesia; 3 Department of Medicinal Chemistry, Faculty of Pharmacy, Universitas Halu Oleo, Kendari 93231, Indonesia; 4 Department of Pharmacy, Universitas Mandala Waluya, Kendari 93231, Indonesia

**Keywords:** alopecia, enzyme 5-α-reductase, molecular docking, molecular dynamics, ADME-Tox, *Merremia peltata*, scopolin, scopoletin

## Abstract

Alopecia is a condition in which hair on the scalp or other areas of the body is lost or falls out excessively. Nutritional deficiency causes blood flow to the head to decrease causing the hormone testosterone to be changed by the enzyme 5-α-reductase to dihydrotestosterone, which inhibits the growth phase and accelerates the death phase. One of the methods developed to treat alopecia is through inhibition of the 5-α-reductase enzyme, which converts testosterone to its more potent metabolite, dihydrotestosterone (DHT). Ethnomedicinally, *Merremia peltata* leaf is used by the people of Sulawesi as a remedy for baldness. Therefore, in this research, an in vivo study was conducted on rabbits to determine the anti-alopecia activity of *M. peltata* leaf compounds. The structure of the compounds isolated from the *M. peltata* leaf ethyl acetate fraction was determined by analysis of NMR and LC-MS data. An in silico study was then carried out using minoxidil as a comparison ligand; scopolin (**1**) and scopoletin (**2**) isolated from *M. peltata* leaf were identified as anti-alopecia compounds by predicting docking, simulating molecular dynamics and predicting absorption, distribution, metabolism, excretion, and toxicology (ADME-Tox). Compounds **1** and **2** had a better effect on hair growth compared to positive controls, and NMR and LC-MS analysis showed that they had comparable binding energies to receptors in the molecular docking interaction study: −4.51 and −4.65 kcal/mol, respectively, compared to −4.8 kcal/mol for minoxidil. Molecular dynamics simulation analysis with the parameters binding free energy calculated using the MM-PBSA method and complex stability based on SASA, PCA, RMSD, and RMSF showed that scopolin (**1**) has a good affinity for androgens receptors. The ADME-Tox prediction for scopolin (**1**) showed good results for the parameters of skin permeability, absorption and distribution. Therefore, scopolin (**1**) is a potential antagonist to androgen receptors and could be useful in the treatment of alopecia.

## 1. Introduction

Hair loss, in medical terms, is known as alopecia. When it affects the scalp and spreads to other parts of the body, alopecia can render hair loss of function [1,2]. Around 35 million men and 21 million women worldwide reported having alopecia in 2014, and 66% of men and 40% of women who have the condition are over 35 years of age. Genetic, environmental, and nutritional factors can all cause alopecia. Such nutrients as proteins and vitamins, including vitamins A, D, E, and B12 are important nutrients for maintaining healthy hair [2]. Alopecia can be caused by the presence of the 5-α-reductase enzyme, which can produce dihydrotestosterone. The activity of testosterone, which is important for the anagen phase of the hair growth cycle, due to the presence of the 5-α-reductase enzyme, reduces the structure of testosterone to dihydrotestosterone, which causes alopecia. When testosterone’s initial aliphatic ring contains a vinyl group, 5-α-reductase lowers it, forming one bond at the Cα position that forms the material known as dihydrotestosterone [3]. The pathogenetic mechanisms underlying androgenic alopecia are not fully understood. Androgen target tissues include skin and the pilosebaceous unit. The major androgen in circulating, testosterone, can be locally converted by the enzyme steroid 5-α-reductase in dermal papilla cells (DPCs) to dihydrotestosterone. Dihydrotestosterone is ten times stronger than testosterone, causing slow hair growth and altered hair cycles, where the anagen duration shortens in each cycle while the telogen duration usually stays the same or it lengthens [4]. Androgens influence human skin functions, including wound healing, sebaceous gland development, and hair growth [5]. In androgenetic alopecia hair follicles, dihydrotestosterone binds to androgen receptors, causing miniaturization of hair follicles and reducing the period of the anagen phase [6,7]. The activity of steroid 5α-reductase types 1 and 2 in bald hair follicles was higher than in non-bald hair follicles [8]. One way to prevent alopecia is by using the synthetic drugs minoxidil and finasteride, but their continuous use can cause other diseases: minoxidil has the side effect of hypertrichosis and the finasteride can reduce libido [9]. Alopecia can be treated in several alternative ways than synthetic medications, one of which is by using the active compounds found in the Indonesian plant *Merremia peltata*. According to ethnobotany, this plant has been used by the Konawe people, Kendari city, Southeast Sulawesi, eastern Indonesia to treat dandruff and hair growth [10]. Based on the docking results for androgen receptors, in silico studies have shown that the terpenoid and steroid chemicals found in *M. peltata* leaves exhibit anti-alopecia activity [11]. In vivo studies on the growth of rabbit hair revealed that a 30% concentration of the ethanol extract of *M. peltata* leaf had good effectiveness and activity regarding hair growth. The semipolar portion has the most active behavior. The vacuum liquid chromatographic fraction of the LC-MS study of *M. peltata* leaves revealed the presence of steroid compounds, flavonoids, coumarins, and aromatic polyketides. The results of in silico tests carried out on vacuum liquid chromatographic fractions of *M. peltata* leaves revealed that steroid molecules, flavonoids, coumarins, and aromatic polyketides at the androgen receptor 4K7A provided better binding energy [12].

Based on previous research, in this study, compounds were isolated from a semipolar fraction of *M. peltata* leaves using radial chromatography (KR) with a separation technique based on centrifugal force, aiming to determine the anti-alopecia activity of the isolated compounds and to characterize them using proton and carbon nuclear magnetic resonance (NMR). Molecular interactions and interaction stability between isolated compounds and the target protein were analyzed using molecular docking and molecular dynamics (MD).

## 2. Results

### 2.1. Elucidation of the Compounds Structure

**Figure 1:**
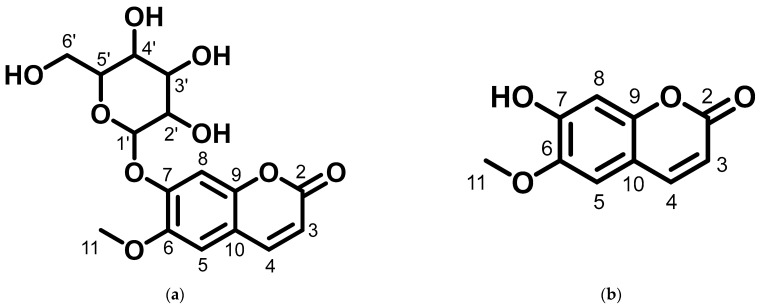
Structure of (**a**) compound **1** and (**b**) compound **2** from the leaf of *M. peltata*.

The results showed that compounds **1** and **2**, isolated from *M. peltata* leaf, are coumarin derivatives. Compound **1** was obtained in the form of a white amorphous powder and, as deduced from its HR-ESI-TOF mass spectrum (*m*/*z* [M + H]^+^ 355.1055), fragmentation analysis showed the presence of a weight m/z 193.0523, indicating a fragment pattern of the glucose group in the form of an aliphatic ring. IR analysis of the functional groups in compound **1** revealed the presence of a hydroxyl group (3440 cm^−1^), a carbonyl group (1718 cm^−1^), and an aromatic group (1574–1485 cm^−1^) [13]. The NMR data (Table 1) scopolin compound showed characteristics for a coumarin derivative on the ^13^C-NMR spectrum of 16 carbon atom signals, including a carbonyl signal (C=O) at δC 163.5 ppm, which is typical of an aliphatic ring that undergoes an enolization reaction at carbon number 3 to coumarin compounds, and there are 4 other quaternary carbon signals (Cq) that have chemical shifts with respective values of δC 151.8, δC 150.6, δC 148.3, and δC 110.7 ppm. Furthermore, there is a signal of methoxy carbon (C-O) at a shift of δC 57.0 ppm. Next, the signals at δC 114.5, δC 145.6, δC 105.1, and δC 102.0 ppm are aromatic carbon signals, each representing one methine carbon (C-H) with a shift in position, indicating a group of coumarin compounds. There is a typical signal for the glucoside group attached to the aromatic ring at the shifts of δC 78.4, δC 77.8, δC 74.7, and δC 71.1 ppm. There is also a signal at the δC 62.4 shift, indicating the presence of a methylene group on the glucoside and at the δC 113.9 shift in the glucoside ring with positions that interact directly with the electron withdrawing group in the form of oxygen [14]. In the ^1^H-NMR data, the singlet at δH 3.90 ppm indicates three protons of the methoxy group (H-11). The following doublets at 6.19 ppm (1H, *J* = 11.5 Hz) and 7.85 ppm (1H, *J* = 11.5 Hz) confirm the presence of H-3 and H-4 protons of the coumarin skeleton. Singlets at chemical shift values of 6.76 ppm and 7.10 ppm reveal two aromatic protons at positions: H-8 and H-5. Furthermore, The following triplet at 3.55 ppm (1H, *J* = 9 Hz), 3.48 ppm (1H, *J* = 9 Hz), and 3.43 ppm (1H, *J* = 9 Hz) confirm the presence of H-2′, H-3′, and H-4′ protons of the glucoside skeleton. One doublet signal on a chemical shift of 5.26 ppm (1H, *J* = 7.5 Hz) at position (H-1′) and one chemical shift of 3.22 ppm, which has a multiplet multiplicity on (H-6′), respectively [15]. The structure of scopolin determined as a result of ^1^H and ^13^C NMR analysis can be seen in Figure 1.

Compound **2** was obtained in the form of yellow amorphous crystals and, as deduced from its HR-ESI-TOF mass spectrum (*m*/*z* [M + H]^+^ 192.0422), fragmentation in compound **2** shows a fragmentation pattern on the hydroxyl group and carbonyl group, evidenced by the presence of bands at *m*/*z* 178.0253 and *m*/*z* 150.0303 [16] in the spectral data; IR analysis revealed the presence of a hydroxyl group (3334 cm^−1^), a carbonyl moiety (1702 cm^−1^), and an aromatic ring (1628 cm^−1^). The characteristics of coumarin derivatives are evident in the ^13^C-NMR spectrum of the scopoletin compound, as shown in Table 1. The spectrum contains 10 signals for carbon atoms, including a carbonyl signal (δC (C=O)) at 164.2 ppm on an aromatic ring that is located next to an electron-withdrawing group in the form of oxygen. In addition, there are 4 other Cq signals that shifted values, δC 152.9, δC 151.4, δC 147.3, and δC 112.4 ppm, respectively. Furthermore, the signal of the methoxy carbon in the aromatic ring is at a shift of δC 56.7 ppm. Next, the signals at δC 113.0, δC 146.1, δC 109.9, and δC 104.0 ppm are aromatic carbon signals, each representing one methine carbon (C-H) with a shift in position, indicating the group of coumarin compounds [14]. In the ^1^H-NMR data, the singlet at δH 3.87 ppm indicates three protons of the methoxy group (H-11). The following doublets at 6.14 ppm (1H, *J* = 11.5 Hz) and 7.81 ppm (1H, *J* = 11.5 Hz) confirm the presence of H-3 and H-4 protons of the coumarin skeleton. Two singlets at chemical shift values of 6.76 ppm and 7.16 ppm reveal two aromatic protons at positions: H-8 and H-5, respectively [17]. The structure of scopoletin determined as a result of ^1^H and ^13^C NMR analysis can be seen in Figure 1. Data from ^1^H and ^13^C NMR analysis for compounds **1** and **2** are shown in Table 1.

The structure of compound **1** and **2** determined as a result of ^1^H and ^13^C NMR analysis can be seen in Figure 1.

Based on the ^1^H and ^13^C spectral data for compounds **1** and **2**, there are the chemical shift values that are not much different for carbon atoms 2 to 11 showing that compounds **1** and **2** have the same basic structure, in the form of a coumarin compound, which is substituted for a methoxy group on carbon number 11.

### 2.2. Docking Simulation of Minoxidil, Finasteride, and Test Ligands (Scopolin and Scopoletin from M. peltata Leaf)

#### 2.2.1. Preparation of Protein Receptor

DHT receptors or 5-α-reductase enzymes can be selected to determine anti-alopecia activity. The selection of the androgen receptor in this study was based on the dihydrotestosterone content of the receptor, which plays a role in shortening the growth phase and accelerating the death phase of hair [18,19]. In bald scalp, genetically programmed miniaturization of hair follicles is controlled by increased uptake, metabolism, and conversion of testosterone to dihydrotestosterone (DHT) with the help of the enzyme 5-α-reductase type II. Steroid 5-α-reductase type II is responsible for 2/3 of circulating DHT causing alopecia [20,21]. The androgen receptor is a protein of the nuclear receptor type whose activity can be activated by formation bond interactions with androgens. Androgen receptors are known as NR_3_C_4_ (nuclear receptors subfamily 3, group C, member 4), which functions as transcription factor on regulation specific gene expression development of the male sex phenotype [12].

#### 2.2.2. Validation of Molecular Docking Method

The Discovery Studio Visualizer software was used to identification the interaction between minoxidil and the androgen receptor (4K7A). The results of the analysis of the binding energy of minoxidil −4.8 kcal/mol showed an RMSD value of 2.31, with the interaction between hydrogen bonds and amino acids, namely SER^865^ and GLU^793^, which have bond values of 2.28 and 2.90 Ǻ [12], respectively.

**Figure 2:**
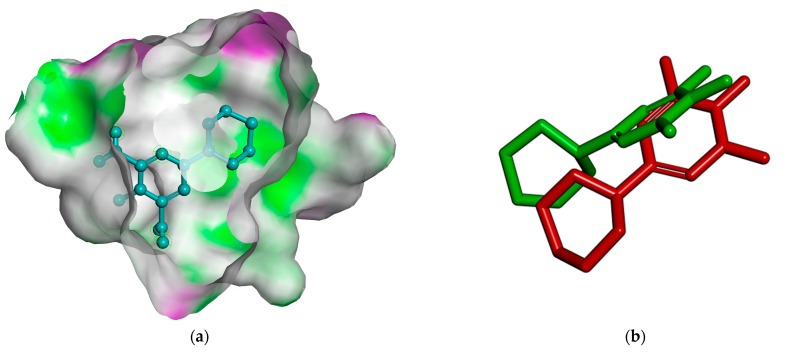
(**a**) Representation of the interactions between the androgen receptor and minoxidil (4K7A). White clouds are hydrophobic interactions and (**b**) overlay of docked pose of minoxidil with that of the co-crystallized ligand of 4K7A. Natural ligand minoxidil (green) before docking and natural ligand minoxidil (red) after docking.

Hydrogen bonds originating from the amino acids SER^865^ and GLU^793^ are interactions that occur between minoxidil and the receptor. The closest residues are LEU^862^, LYS^861^, and TYR^857^, which are present in the androgen and minoxidil receptor complexes (Figure 2).

#### 2.2.3. Docking Simulation

The docking simulation was carried out using the AutoDock Tools 1.5.6 (Scripps Research Institute, San Diego, CA, USA) program by selecting the coordinates that identify the position of the androgen receptor’s interaction to minoxidil (4K7A). Analysis was carried out to determine the value of ΔG, and the hydrogen bonds formed with each of the minoxidil, finasteride, and two test ligands whose structures were obtained from the results of analysis using NMR and LC-MS.

The docking simulation results for the natural minoxidil ligand obtained a binding energy value of −4.8 kcal/mol with a hydrogen bond distance obtained of 2.28 and 2.90 for the amino acid SER^865^ and GLU^793^ while the standard compound finasteride had a binding energy value of −6.03 kcal/mol with a hydrogen bond distance of 2.86, 1.88, and 2.13, which interact with the amino acids ARG^854^, GLU^793^ and SER^865^ and have the nearest amino acid residues LEU^862^, LYS^861^, TYR^857^, LYS^861^, and LEU^797^ [12]. The results of the docking simulation scopolin and scopoletin are shown in Table 2.

**Figure 3:**
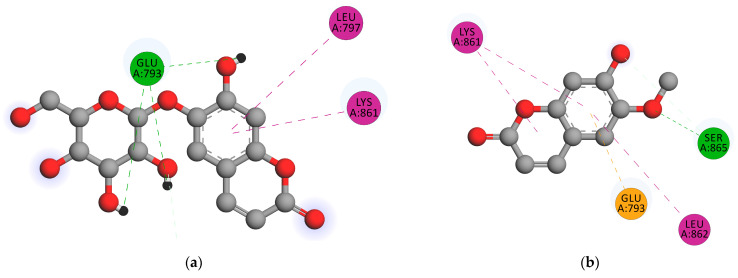
Visualization of androgen receptor docking with (**a**) Scopolin and (**b**) Scopoletin. Hydrogen interactions (green), hydrophobic interactions (purple), and anionic interactions (orange).

A description of the interactions between the androgen receptor (4K7A) with the standard finasteride and the coumarins derived from *M. peltata* leaf was obtained. The ΔG value of scopolin and scopoletin was comparable but lower than that of finasteride. Visualization of the docking results is shown in Figure 3.

#### 2.2.4. Molecular Dynamic Simulation

**Figure 4:**
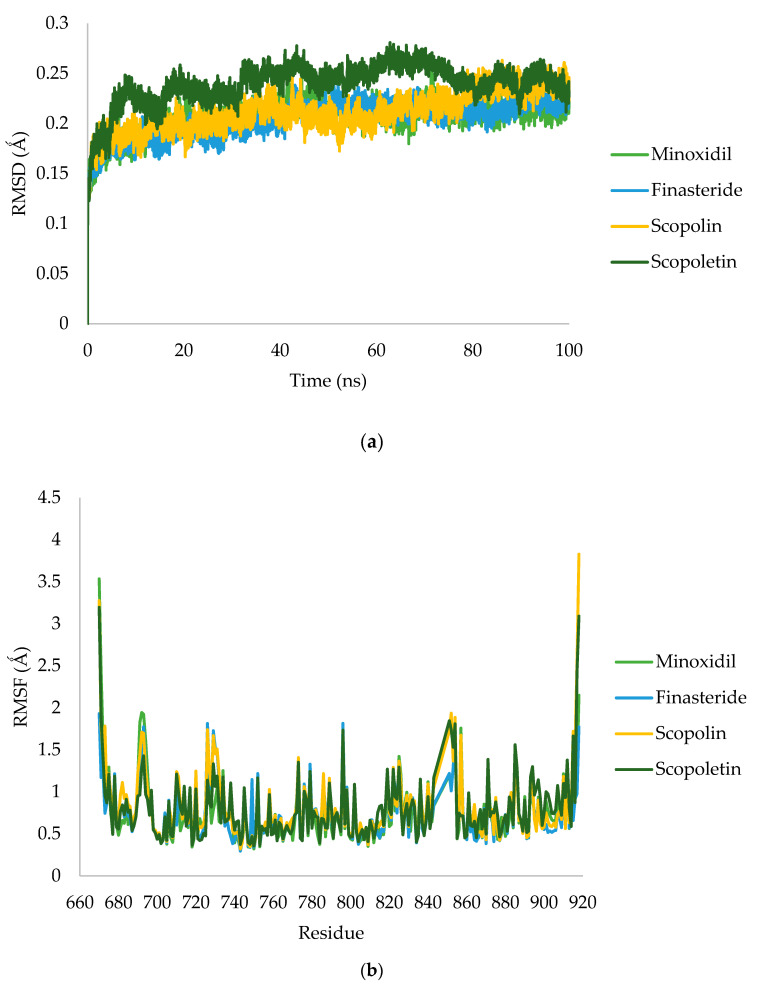
RMSD (**a**) and RMSF (**b**) value of minoxidil (green), finasteride (blue), scopolin (orange), and scopoletin (dark green).

The MD simulation was carried out using the reference chemical finasteride, the natural ligand minoxidil scopolin, and scopoletin, where the two test compounds had the same ΔG value. The RMSD and RMSF analysis of the receptor–ligand complex using GROMACS 2016 was carried out by measuring the stability of the RMSD and RMSF values in the system during the simulation (Figure 4).

**Figure 5:**
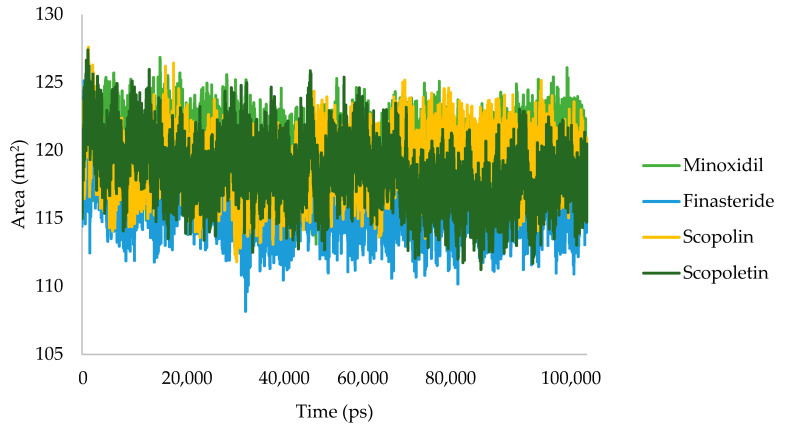
SASA plot of minoxidil (green), finasteride (blue), scopolin (orange), and scopoletin (dark green).

To forecast the conformational changes in proteins that make them accessible to water molecules, the SASA was the result during simulations. Figure 5 illustrates the results of this investigation, which demonstrated that scopoletin was more stable at the androgen receptor than minoxidil.

**Figure 6:**
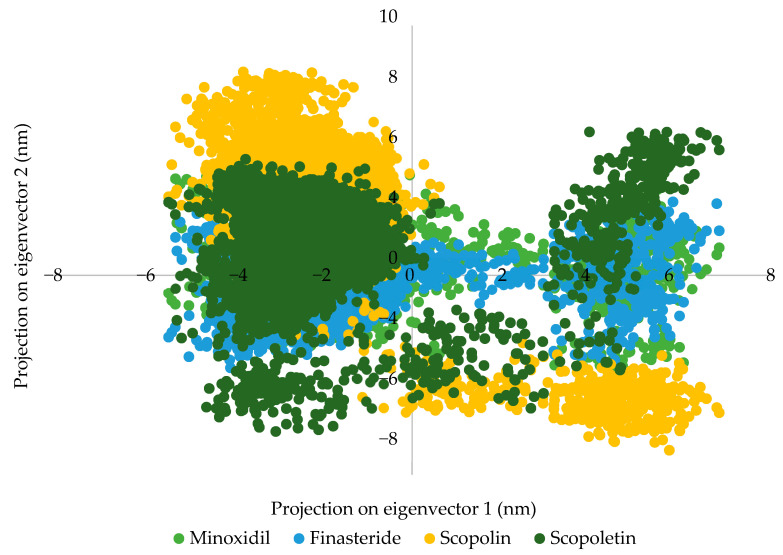
PCA plot of minoxidil (green), finasteride (blue), scopolin (orange), and scopoletin (dark green).

PCA is used to look for significant changes in the protein–ligand complex. The eigenvectors, which control complex motion and dynamics, are investigated for their direction and amplitude in 2D trajectory plots. The stability of scopolin for a 100 ns simulation of its binding to the androgen receptor can be seen by plotting it (Figure 6).

The binding free energy of the MD trajectories of the system complex was calculated using the MM-PBSA method for a timestep of 0–100 ns (Table 3).

#### 2.2.5. ADME-Tox Prediction

Identification of substances that can function as drugs is carried out using pharmacokinetic analysis and prediction of ADME-Tox. Compounds with anti-alopecia activity were analyzed in this study using ADME-Tox analysis; projected values are shown in Table 4 and Table 5.

### 2.3. Hair Growth Activity of Scopolin and Scopoletin from M. peltata Leaf

**Figure 7:**
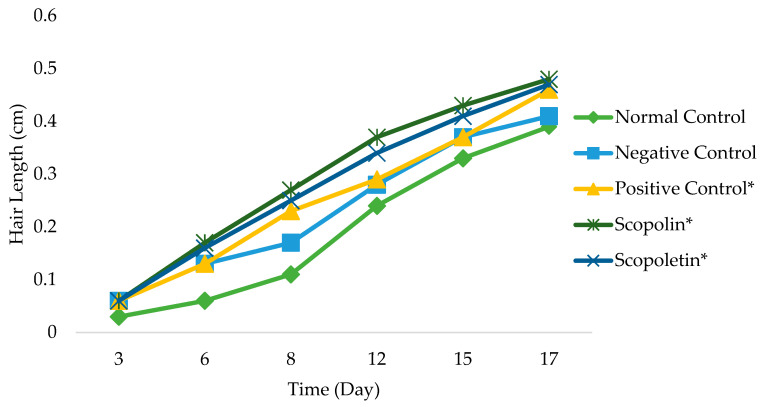
Test of scopolin and scopoletin from *M. peltata* leaf on rabbit hair growth (* show good growth activity).

An in vivo hair growth activity test of scopolin and scopoletin isolated from *M. peltata* leaf at a concentration of 30%, with a positive control of 2% minoxidil, a negative control of Na-CMC, and a normal control, was carried out for 17 days of observation on test animals. On average, the fasting hair growth level was 0.46 ± 0.11 * mg/dL in the positive control group (minoxidil), 0.39 ± 0.04 mg/dL in the normal control group, and 0.41 ± 0.02 mg/dL in the negative control group; it was 0.48 ± 0.07 * and 0.47 ± 0.03 * mg/dL for scopolin and scopoletin, respectively Figure 7.

## 3. Discussion

### 3.1. Docking Simulation of Minoxidil, Finasteride, and Test Ligands (Scopolin and Scopoletin from M. peltata Leaf)

#### 3.1.1. Preparation of Protein Receptor

The binding interactions with androgens activate the androgen receptors. Physiologically, this hormone exists in both sexes, but are more abundant in males because they serve as a precursor to the female sex hormones that are then transformed into estrogen [22]. The androgen receptor model with PDB code 4K7A is used in this investigation. Residues 690 to 919 code for the androgen receptor’s ligand-binding domain (unitprot.org).

An essential component of docking is a ligand. The Lipinski rule is the initial parameter chosen in the ligand selection procedure used in target protein tethering. With a binding energy (ΔG) of about −4.8 kcal/mol, minoxidil interacted with the androgen receptors SER^865^ and GLU^793^ through 2.28 and 2.90 Å hydrogen bonds after being prepared as a natural ligand (4K7A). A more stable bond is indicated by a lower ΔG value.

#### 3.1.2. Validation of Molecular Docking Method

Redocking of minoxidil to a target protein is a molecular validation of the docking involving minoxidil in its initial state dissociated with a resolution value of 2.44 Å on the androgen receptor (4K7A) as the initial coordinate. The ΔG value obtained in the docking process of the natural minoxidil ligand was −4.8 kcal/mol, with the RMSD value of minoxidil redocking being 2.31. According to previous research [23], RMSD values of 3 Å and ΔG, which are the same as the results of redocking, indicate that the interaction between the ligand and the receptor is in a low energy state, which causes the molecule to become more stable. Hydrogen bonds on SER^865^ and GLU^793^ towards the -NH_2_ and -NO functional groups on minoxidil showed values of 2.28 and 2.90, respectively. The interaction between SER^865^ and GLU^793^ became the binding domain between the ligand and the androgen receptor.

#### 3.1.3. Docking Simulation

The docking method requires a binding interaction between ligand and protein, which is used to predict the position and orientation of the ligand when it binds to the protein receptor. The ΔG value assigned during the docking process is an indicator of conformational stability between the ligand and the androgen receptor [24]. Based on the results of docking minoxidil to the androgen receptor, the ΔG value for minoxidil was −4.8 kcal/mol (Table 2), −4.51 kcal/mol for scopolin, and −4.65 kcal/mol for scopoletin.

The interactions between the amino acids SER^865^ and GLU^793^ that result in the hydrogen bonds between minoxidil and the androgen receptor contain the closest amino acid residues, LEU^862^, LYS^861^, and TYR^857^. The docking results showed that scopolin and scopoletin had ΔG that were almost the same as that of minoxidil. The ΔG of scopoletin is affected by the presence of the same amino acid in minoxidil for the hydrogen bonds formed, namely SER^865^, and this amino acid is also present in the standard finasteride. The amino acid residues involved in forming hydrogen bonds in scopolin are LYS^861^ and LUE^797^ (Figure 3), whereas scopolin also has GLU^793^. The presence of the same amino acid as in minoxidil and finasteride causes this compound scopolin to have a ΔG that is almost the same as the natural ligand minoxidil. The ΔG of scopolin, scopoletin, and finasteride are nearly the same as that of minoxidil because they are influenced by the presence of hydrogen bonds formed with the amino acids SER^865^ and GLU^793^ with interaction values of 2.28 and 2.90 Å, respectively. The interactions that occur are hydrogen bonds, hydrophobic interactions, and electrostatic interactions on an anchorage area < 5Å. Hydrogen bonds involve the interaction between covalently bonded hydrogen atoms and electronegative atoms such as oxygen [25]. Scopolin forms hydrogen bonds through electrostatic interactions with a hydrogen bond distance of 1.86 Å with the amino acid GLU^793^, which interacts with the hydroxyl -OH group, and scopoletin forms hydrogen bonds with a value of 1.80 Å with the amino acid SER^865^, which interacts with the methoxy group. Electrostatic interactions also play a role in the stability of binding of the ligand to the receptor. Electrostatic interactions are interactions between atoms that occur due to differences in the polarity of the ligand and the receptor [26]. Hydrogen bonds formed with short bond distance indicate a more stable bond strength than those with longer bond distances because the hydrogen bond distance determines the stability of binding of the ligand to the androgen receptor [27]. Interactions known as hydrophobic interactions avoid liquid environments and frequently gather near the globular shape of proteins [28]. The formation of hydrophobic interaction can minimize the interaction between nonpolar residues and water. In minoxidil, the hydrophobic interaction with the ligand occurs at residues LEU^862^, LYS^861^, and TYR^857^, while in scopoletin, it involves residues LEU^862^, LYS^861^, and GLU^793^, and in scopolin it involves residues LYS^861^, LUE^797^, and LEU^862^. Residues involved in hydrophobic interactions are nonpolar amino acid residues that tend to form groups on the interior of the protein.

The residues SER^865^ and GLU^793^ are projected to play an important role in the agonist receptor binding region as they are consistently present in all receptor–ligand interactions in Table 3 as hydrogen bonds or hydrophobic interactions. Based on the simulation findings using natural ligands, GLU amino acids are projected to play an important role in the region of receptor protein binding sites. These amino acids are found in hydrogen bonds from interactions with receptors. A protein binding site is a region of the protein where molecules and ligands are bound, which affect the shape and function of the protein. Amino acid residues that play an important role in interactions with ligands are found in the binding site area. Amino acid residues, which play an important role in acting with the ligands, present in the region of the binding site. The interactions formed between the ligands and the amino acid residues are hydrogen bonds, hydrophobic interactions, and electrostatic interactions.

According to Kendel et al., seven substituted coumarin derivatives have been shown to be potentially useful androgen receptor antagonists [29]. According to Koga et al., selected coumarin amide derivatives showed androgen receptor antagonist activity against LNCaP cells with the T877A androgen receptor and had weak prostate androgen antagonist activity [30]. Another study carried out by Morikawa et al. regarding the inhibitory activity of the testosterone 5-reductase derivatives of geranylated coumarin surangin C and mammea A/AB cyclo D isolated from the methanol extract of *Mammea siamensis* flowers showed that the geranylated coumarin derivatives provided fairly good inhibitory activity [31].

#### 3.1.4. Molecular Dynamic Simulation

The stability of the complex was evaluated using RMSD analysis, while the stability of individual amino acid was evaluated using RMSF analysis. The analysis was performed by comparing the two reference ligands and the test ligands that exhibit androgen receptor inhibition of minoxidil and finasteride. Scopolin showed the highest metabolite docking score, as simulated during MD analysis, and had complex stability. In contrast to scopolin, which exhibits the same fluctuations as minoxidil and finasteride in complexes with androgen receptors, scopolin exhibits high fluctuations. Finasteride, scopolin, scopoletin, and minoxidil each had an average RMSD fluctuation of 0.206, 0.203, 0.211, and 0.239, respectively. This shows that finasteride and scopolin show the lowest fluctuations compared to natural ligands indicating that these ligands have reached a stable conformation that binds to proteins [32]. RMSF-measured amino acid fluctuations of the two receptor complex systems showed a consistent pattern in all regions. The androgen receptor shows greater fluctuation than the other residues at residues 693, 726, 729, and 796. These residues are the amino acids responsible for the loop regions in the protein structure.

Figure 5 shows the results of the SASA analysis for the MD trajectory at 100 ns simulation. Scopolin and scopoletin in the graphs show similar changes on SASA analysis to the larger androgen receptor ligand complex than minoxidil and finasteride. Minoxidil, finasteride, scopolin, and scopoletin had average values of 120.13, 115.93, 118.43, and 119.13 nm^2^, respectively. A low SASA score indicates a complex system that is becoming more stable [32]. This analysis is in agreement with RMSD values, indicating that finasteride has greater androgen receptor stability than minoxidil, scopolin, and scopoletin.

Figure 6 shows the two eigenvector projections for finasteride, minoxidil, scopolin, and scopoletin in complex with androgen by selecting PC1 and PC2, respectively. Groups occupying more space represent a more stable molecule, while groups occupying less space represent less stable protein–ligand interactions [32]. The 2D eigenvector plots in the figure show that scopolin occupies less space phase compared to minoxidil and finasteride. Analysis showed that scopolin remained stable for 100 ns during simulated androgen receptor binding.

Polar solvation energy has a positive value while van der Waals, electrostatic, and SASA energies have a negative value in both of these complex systems. The results show that van der Waals, electrostatic, and SASA energy favor the binding while polar solvation energies oppose it in both complex systems. The total binding free energy of the ligands had varying values. Finasteride provided the lowest binding free energy −62.867 kJ/mol, while those for minoxidil, scopolin, and scopoletin were −51.810, −37.404, and 269.626 kJ/mol, respectively. The MM-PBSA analysis indicated that scopolin has better affinity for the androgen receptor.

#### 3.1.5. ADME-Tox Prediction

PB plasma protein percentage (%) [33] is a significant pharmacokinetic factor affecting dosing frequency but not daily dosing. Distribution is due to changes in plasma protein binding. Pharmacological interactions involving distribution processes are clinically important for a drug if its percentage PB is greater than 85%, its volume of distribution is low, and its margin of safety is very small. The minoxidil PB value was 99%, and volume of distribution was 0.142. The percentage PB value of scopoletin was not good, and the volume of distribution was −0.611, while the percentage PB scopoletin value was 91% and the volume of distribution value was 0.034. These findings indicate that scopoletin and minoxidil have strong plasma protein binding properties. The Vdss reaction volume can be predicted using the predicted distribution values made using the pkCSM tool. More drug reserves from the plasma are delivered to the tissues at higher Vdss values. According to a previous study [34], if the Vdss log value is between <−0.15 and >0.45 then the Vdss value is acceptable with a low distribution volume. According to analysis with pkCSM, the log Vdss value for minoxidil was 0.142, compared to scopolin and scopoletin values of 0.363 and 0.611, respectively. Scopoletin outperformed minoxidil and scopolin in what is considered an acceptable Vdss value [34].

Two in vitro models that can be used to predict oral drug absorption are the Caco-2 and MDCK cell models. In vitro intestinal mucosal models were used to estimate drug absorption based on the permeability of single cell Caco-2 monolayers. Although a higher polar surface area results in a stronger hydrogen bonding interaction, polar binding does not result in good Caco-2 permeability between Caco-2 cells and drugs [35]. A compound is said to have strong Caco-2 permeability if Papp > 8 × 10^6^ cm/s [34]. A score > 0.90 is considered a high Papp log value and indicates that the compound is permeable based on the findings of the pkCSM study. The Papp values for minoxidil, scopoletin, and scopolin in this study were 0.653, 1.184, and 0.377, respectively. This shows that scopoletin has higher Caco-2 permeability than scopolin and minoxidil.

### 3.2. Hair Growth Activity of Compounds Scopolin and Scopoletin Isolated from M. peltata Leaf

Based on a modified method, scopolin and scopoletin were tested for their ability to stimulate hair growth in male rabbits [36]. To protect the safety and rights of test animals, the research ethics committee approved this study (1827/UN29.20.1.2/PG/2020). Adaptation to the environment was carried out to reduce stress on test animals so that rabbits can adapt to their environmental conditions.

After 17 days of testing, areas treated with scopolin and scopoletin at a concentration of 30% showed the highest hair growth rate, with respective values of 0.48 ± 0.07 and 0.47 ± 0.03 mm, while the normal control showed the least hair growth, namely 0.39 ± 0.04 mm. Figure 1 shows that the positive control of minoxidil, scopolin, and scopoletin provided fast hair growth, while normal control and negative control hair growth were not so fast. Alkaloid, pyron, and flavonoid compounds are said to be substances that actively help in hair fertilization process [37]. It is known that derivatives of the flavanone class of flavonoid compounds have a therapeutic effect on hair growth. According to previous research [12], the results of in vivo tests on the secondary metabolites found in *M. peltata* extracts showed that they can increase hair growth.

## 4. Materials and Methods

### 4.1. Materials

Rawa Aopa National Park in Southeast Sulawesi was the site of sampling of M. peltata leaves. The organic solvents used for analysis were technical EtOH, n-hexane, ethyl acetate (EtOAc), acetone, methanol (MeOH), and chloroform (CHCl3) (Darmstadt, Germany). In addition, Merck 60 PF254 silica gel (Darmstadt, Germany) served as the stationary phase in radial chromatography (KR), silica gel Merck 60 (0.2 to 0.5 mm) (Darmstadt, Germany) was used for thin layer chromatography (TLC), and Merck 60 silica gel (0.2 to 0.5 mm) (Darmstadt, Germany) was used for sample absorption. The isolates were analyzed using LCMS/MS (the waters xevo TQD) using a Phenomenex 5μ C8 HPLC column, 150 × 2 mm id connected to a Q-TOF spectrometer (Boston, MA, USA). The isolated compounds were characterized using Bruker Alpha FTIR spectrometer (Boston, MA, USA), Agilent NMR 500 MHz (1H-NMR) and 125 MHz (13C-NMR) Sacramento, CA, USA, and NMR spectrum analysis using Top Spin 3.6.5 academy software (Boston, MA, USA). Minoxidil 5% as a positive control (Surabaya, Indonesia), Na-CMC solution as a negative control (Darmstadt, Germany), and male New Zealand White rabbit test animals are the components used in the in vivo test (Kendari, Indonesia) [38]. Analysis of the results of the in silico test was carried out using Discovery Studio Visualizer software (Sacramento, CA, USA) [39], Auto Dock Tools 1.5.6 (Sacramento, CA, USA) [40], and GROMACS 2016.3 with the AMBER99SB-ILDN (Groningen, The Netherlands) [41] and ADME-Tox force fields from the pkCSM website (Melbourne, Australia) [42].

### 4.2. Sample Preparation and Determination

A plant herbarium *M. peltata* leaf was verified (844/I1.CO2.2/PL/2020) at the School of Life Science Technology, Bandung Institute of Technology, West Java.

### 4.3. Extraction

Dried powder of *M. peltata* leaves was obtained by preparing 1.3 kg of wet sample, which was ground into powder and then heated at 30–40 °C until dry. The extraction process was carried out by maceration method of 1 kg of dry powder using 6 L of 96% ethanol for 3 × 24 h with a ratio (1:2). The ethanol extract of *M. peltata* leaves in viscous form had a mass of 244.86 g.

### 4.4. Extract Purification and Isolation

**Figure 8:**
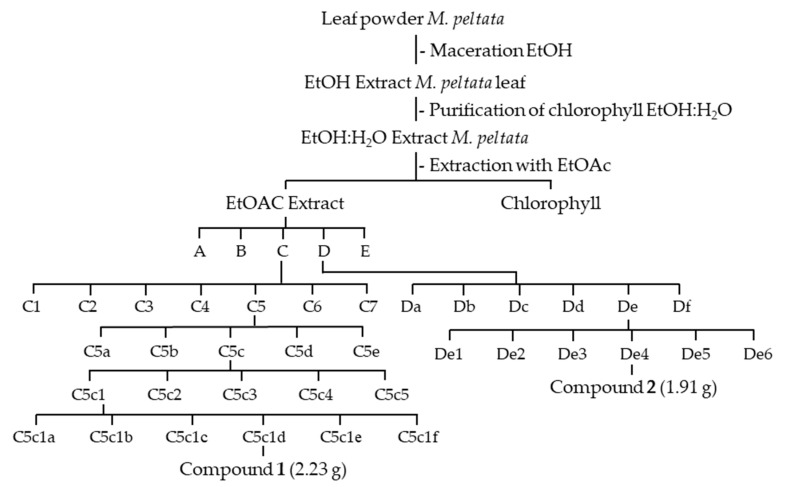
**Figure 8**. Isolation of compounds scopolin and scopoletin from EtOAc extract of *M. peltata* leaf.

We applied a method of separating chlorophyll contained in ethanol extract using precipitation with a 1:1 methanol:water ratio. The precipitate formed after 24 h through the process of mixing and stirring. The filtrate was then separated from the precipitate formed from chlorophyll using ethyl acetate (3L) with a ratio (1:1). The chlorophyll-free filtrate obtained was added by adding MgSO_4_ powder to the ethyl acetate phase, which was then filtered. The solvent for the ethyl acetate phase was evaporated and 40 g of ethyl acetate extract was obtained. Further separation was carried out using TLC at a ratio of n-hexane:ethyl acetate (1:1) as an eluent; the separation pattern of each purified fraction was examined [9]. Five main fractions (A–E) were produced after the ethyl acetate extract was separated with VLC silica gel and eluted with n-hexane:ethyl acetate (10:0–0:10), 100% MeOH to dissolve polar compounds. The C fraction (6.3 g) was then separated by KR using n-hexane:ethyl acetate (8:2) as the eluent, resulting in seven mixed fractions (C1–C7). Five fractions (C5a–C5e) were obtained based on the chromatogram. The C5 fraction (3.71 g) was separated again using the KR method with n-hexane:ethyl acetate (7:3) as the eluent. Further separation of the C5c fraction (2.86 g) with n-hexane:ethyl acetate solvent resulted in five mixed fractions (C5c1–C5c5) (7:3). After further separation by KR to purify the C5c1 fraction using n-hexane:ethyl acetate (7:3) as eluent, six combined fractions (C5c1a–C5c1f) were obtained, and the purity of the C5c1d fraction (2.33 g) was then confirmed using TLC. The purity test was carried out using two eluent systems: n-hexane:ethyl acetate and CHCl_3_:MeOH. Compound **1** is visible on the KR chromatogram of the C5c1d fraction. Six combined fractions (D1–D6) were produced from the D fraction (7.66 g), which was separated by KR using n-hexane:ethyl acetate (1:1) as eluent. Then, the 3.71 g De fraction was separated by KR again, using n-hexane:ethyl acetate (6:4). The five combined fractions resulted from the separated De fractions (De1–De5). Compound **2** was produced after testing its purity on the 2.21 g De4 fraction with TLC by using a comparison of two different eluent systems, namely n-hexane:ethyl acetate and CHCl_3_:MeOH. Figure 8 shows the method for isolating compounds **1** and **2**.

### 4.5. Elucidation of the Compounds’ Structures

The structures of compounds scopolin and scopoletin were determined using spectroscopic techniques including IR spectroscopy, LC-MS, and 1-D NMR (^1^H and ^13^C-NMR with DEPT technique) [43].

### 4.6. Docking Simulation of Minoxidil, Finasteride, and Test Ligands (Compounds Scopolin and Scopoletin from M. peltata Leaf)

#### 4.6.1. Preparation of Ligand Structure

The Table 6. contains the structures of the reference ligands minoxidil, finasteride, and the pure compounds scopolin and scopoletin isolated from the ethyl acetate extract of *M. peltata* leaves, and ChemDraw 8.0 was used to convert the two-dimensional (2D) structure of the test ligands, finasteride, and minoxidil into a three-dimensional structure (3D).

#### 4.6.2. Preparation of Protein Receptor

The crystal structure of the androgen receptor (4K7A) was used with type parameters. The selected species was *Homo sapiens*, and the mutation value was 0. The PDB database used has natural ligands DHT and minoxidil at a resolution of 2.44. The natural ligand present in the androgen receptor is minoxidil. The drug minoxidil is used to treat baldness caused by DHT. A PDB file containing the 3D structure of the androgen receptor protein (4K7A) and its organic ligands was created using AutoDock Tools 1.5.6 [40]. The grid box’s position was used for determining the docking material’s spatial geometry and coordinates. Docking studies of all the test ligands used the grid box location of minoxidil in the androgen receptor crystal structure.

#### 4.6.3. Validation of the Molecular Docking Method

Molecular docking validation using AutoDock Tools 1.5.6 (Scripps Research Institute, San Diego, CA, USA) was conducted by redocking the modified natural ligand against the target protein. To obtain the appropriate grid position, the natural minoxidil ligand is reattached to the 4K7A androgen receptor in its previous position. The receptor protein contains residues in the form of DHT, minoxidil, and water ligands, which are then removed by moving the grid box to the natural minoxidil ligand. Natural ligand redocking is considered effective if the RMSD value < 3 Å [23]. This process was used to calculate the RMSD value using the Discovery Studio Visualizer program to examine the overlay of the separated natural minoxidil ligands and the reassembled minoxidil ligands and obtained an RMSD value of 2.31 Å.

#### 4.6.4. Docking Simulation

Minoxidil, finasteride, and the test ligands (scopolin and scopoletin), respectively, had optimized 3D structures using Chem3D Ultra 8.0 and MM2 semi-empirical computational methods. By converting the geometry to the smallest potential energy of the 3D structure, the calculation is complete.

The tether coordinates (grid center) for x, y, and z = 40 are used to tether each ligand to the androgen receptor in the pdbqt format, along with the grid box size coordinates of x = 2.592, y = 0.864, and z = 6.729. Each ligand has rigid interactions with biomacromole-cules and is in a stable state.

Analysis of interactions formed from docking results such as hydrogen bonds, hydrophobic interactions, and bond distances, which can be analyzed using the Discovery Studio Visualizer [39].

#### 4.6.5. Molecular Dynamic Simulation

Scopolin and scopoletin were isolated from the EtOAc fraction of *M. peltata* and used as starting material for MD simulations to determine the ligand with the lowest binding energy at the androgen receptor [44]. Ligand and topology parameters were determined using ACPYPE [45]. To calculate the electrostatic force at a certain distance, the Ewald particle mesh method is used [46]. Enter Cl^−^ and Na^+^ ions to neutralize the system. The solution is constructed using the TIP3P water cube model [47]. The minimization process, 310 K heating, temperature acclimatization, and pressure acclimatization are all part of the simulation setup steps. A 2 fs timestep MD stage is produced in 100 ns. The magnitude and direction of the principal movement are estimated using the principal component analysis (PCA) method. By estimating the binding free energy by using the MM-PBSA method, solvent accessible surface area (SASA), and RMSD root mean squared (RMSF) fluctuations from the docking, a post-MD simulation analysis was completed.

#### 4.6.6. Prediction of ADME-Tox

Analysis with pkCSM was used to predict ADME-Tox SAE [42]. PubChem is used to determine the chemical structure, which is then converted into SMILE format. The SMILE canonical structure was downloaded for analysis.

### 4.7. Hair Growth Activity Assay

Scopolin and scopoletin isolated from *M. peltata* leaves were test for their ability to increase hair growth at a concentration of 30% dissolved in Na-CMC solution. Clean-shaving rabbit backs and then cutting six 2 cm × 2 cm squares is one of the methods used in this test to measure hair growth activity. Six healthy male rabbits were used in the test (4–5 months old, without any anatomical defects). Rabbits acclimatized for one week before use. The back hair of each rabbit was then removed and allowed to rest for 24 h. The test material was then applied to the test area once a day for 17 days of treatment. Day 0 of application is the first day [48]. Two-step testing was carried out. The first step was to determine the best extract concentration and effective dosage for rabbit hair growth. Five subsets of the test area were used: the positive control, which was treated with 2% minoxidil; the negative control, which was treated with Na-CMC; the normal control, which received no treatment; and the test group, which received 30% of the scopolin and scopoletin compounds. Data processing was carried out using (ANOVA) after the report data was obtained and the LSD (least significant difference) test was carried out to see the activity of each concentration group, which is significant in hair growth [49].

## 5. Conclusions

Based on in vivo results on rabbit hair growth activity against compounds isolated from *M. peltata* leaves, compounds **1** (scopolin) and **2** (scopoletin) provide excellent hair growth effectiveness, and in silico analysis revealed that scopolin and scopoletin have binding energy that is nearly equal to minoxidil as a natural ligand. However, molecular dynamics simulations show that scopolin has a good interaction with receptors over time compared to the natural minoxidil ligand and the MM-PBSA value of scopolin is close to that of minoxidil. ADME-Tox analysis of scopolin shows a good value profile and in vivo test, in which, at a concentration of 30%, the scopolin compound provided excellent hair growth activity in rabbits. Therefore, the scopolin compound has the potential to be further developed as an anti-alopecia drug. Furthermore, further research is needed to formulate scopolin—which has anti-alopecia properties—in a tonic dosage form and conduct in vivo testing.

## Figures and Tables

**Table 1:** Chemical shift, constant coupling, and multiplicity ^1^H NMR (500 MHz) and ^13^C NMR (125 MHz) data of compounds **1**–**2** in Aceton-*d_6_*.

No	Compound 1		Compound 2	
	δ ^1^H (500 MHz)	δ ^13^C (125 MHz)	δ ^1^H (500 MHz)	δ ^13^C (125 MHz)
2	-	163.5	-	164.2
3	6.19 (1H, *d*, *J* = 11.5)	114.5	6.14 (1H, *d*, *J* = 11.5)	113.0
4	7.85 (1H, *d*, *J* = 11.5)	145.6	7.81 (1H, *d*, *J* = 11.5)	146.1
5	7.10 (1H, *s*)	105.1	7.16 (1H, *s*)	109.9
6	-	150.6	-	151.4
7	-	148.3	-	147.3
8	6.76 (1H, *s*)	102.2	6.76 (1H, *s*)	104.0
9	-	151.8	-	152.9
10	-	110.7	-	112.4
11	3.90 (3H, s)	57.0	3.87 (3H, *s*)	56.7
1′	5.26 (1H, *d*, *J* = 7.5)	113.9		
2′	3.48 (1H, *t*, *J* = 9)	74.7		
3′	3.43 (1H, *t*, *J* = 9)	77.8		
4′	3.55 (1H, *t*, *J* = 9,5)	71.1		
5′	3.58 (1H, *dd*, *J* = 12; 5.5)	78.4		
6′	3.22 (2H, *m*)	62.4		

**Table 2:** Docking simulation results.

Compound	Binding Energy (kcal/mol)	Hydrogen Bond Distance (Ǻ)	Hydrogen Bonds	Nearest Amino Acid Residue(s)
Scopolin	−4.51	1.86	GLU^793^	LYS^861^, LUE^797^, LEU^862^
Scopoletin	−4.65	1.80	SER^865^	LEU^862^, LYS^861^, GLU^793^

**Table 3:** MM-PBSA energy summary ligand–androgen receptor during 500 ns simulation.

Ligand	van der Waals Energy (KJ/mol)	Electrostatic Energy (KJ/mol)	Polar Solvation Energy (KJ/mol)	SASA Energy (KJ/mol)	Total Binding Energy (KJ/mol)
Minoxidil	−134.036 +/− 9.802	−9.222 +/− 12.200	104.959 +/− 13.690	−13.511 +/− 0.592	−51.810 +/− 14.266
Finasteride	−112.958 +/− 17.378	−38.599 +/− 12.965	102.071 +/− 23.052	−13.380 +/− 1.690	−62.867 +/− 14.004
Scopolin	−96.419 +/− 15.585	−100.845 +/− 23.749	171.825 +/− 29.130	−11.966 +/− 1.147	−37.404 +/− 16.986
Scopoletin	−16.785 +/− 34.408	−4.731 +/− 12.402	293.019 +/− 1393.615	−1.878 +/− 3.788	269.626 +/− 1398.112

**Table 4:** Absorption and distribution.

Compound	Absorption				Distribution			
	1	2	3	4	5	6	7	8
Minoxidil	−2.871	0.653	94.641	−2.798	0.142	0.773	−0.951	−3.471
Finasteride	−5.148	1.269	93.742	−3.463	−0.185	0.01	−0.18	−1.821
Scopolin	−2.21	0.377	48.119	−2.822	−0.611	0.397	−1.286	−3.954
Scopoletin	−2.504	1.184	95.277	−2.944	0.034	0.363	−0.299	−2.32

Model name and units: 1—water solubility (numeric log mol/L); 2—Caco-2 permeability (numeric log Papp in 10^−6^ cm/s); 3—intestinal absorption (human) (numeric (% absorbed)); 4—skin permeability (numeric (log Kp)); 5—Vdss (human) (numeric (log L/kg)); 6—fraction unbound (human) (numeric (Fu)); 7—BBB permeability (numeric (log BB)); 8—CNS permeability (numeric (log PS)).

**Table 5:** Metabolism, excretion, and toxicity.

Compound	Metabolism		Excretion	Toxicity							
	9	10	11	12	13	14	15	16	17	18	19
Minoxidil	No	No	0.275	No	−0.359	No	No	2.286	Yes	No	3.516
Finasteride	Yes	Yes	0.38	No	−1.355	No	No	2.424	Yes	No	0.638
Scopolin	No	No	0.716	No	0.393	No	No	3.756	No	No	4.198
Scopoletin	No	No	0.73	No	0.614	No	No	1.378	No	No	1.614

Model name and units: 9—CYP3A4 substrate (yes/no); 10—CYP2C9 inhibitor (yes/no); 11—total clearance (log mL/min/kg); 12—AMES toxicity (yes/no); 13—maximum tolerated dose (human) (log mg/kg/day); 14—hERG I inhibitor (yes/no); 15—hERG II inhibitor (yes/no); 16—oral rat acute toxicity (LD) (mol/kg); 17—hepatotoxicity (yes/no); 18—skin sensitization (yes/no); 19—minnow toxicity (log mM).

**Table 6:** Two-dimensional structure of minoxidil, finasteride, and assay ligands from purified *M. peltata* leaf extract.

No.	IUPAC Name	Structure
1.	Natural ligand Minoxidil	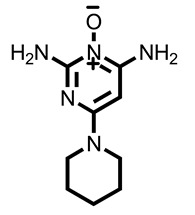
2.	Reference ligand finasteride	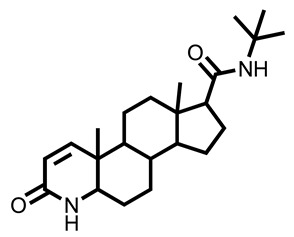
3.	Scopolin	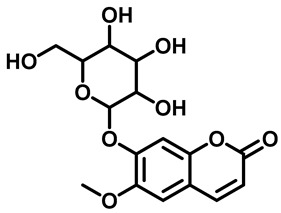
4.	Scopoletin	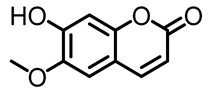

## Data Availability

Not applicable.

## Abbreviations

DHT	Dihydrotestosterone
NMR	Nuclear magnetic resonance
LC-MS	Liquid chromatography mass spectrometry
ADME-Tox	Absorption, distribution, metabolism, excretion, and toxicology
MM-PBSA	Molecular mechanics/Poisson–Boltzmann surface area
SASA	Solvent accessible surface area
PCA	Principal component analysis
RMSD	Root mean square deviation
RMSF	Root mean square fluctuation
DPCs	Dermal papilla cells
VLC	Vacuum liquid chromatography
KR	Radial chromatography
TLC	Thin layer chromatography
MD	Molecular dynamics
IR	Infrared
HR-ESI-TOF	High-resolution electrospray ionization time-of-flight
NR_3_C_4_	Nuclear receptor subfamily 3, group C, member 4
Na-CMC	Carboxymethyl cellulose sodium
DBE	Double bond equivalence
LNCaP	Lymph node carcinoma of the prostate
DEPT	Distortion-less enhancement by polarization transfer
ANOVA	Analysis of variances
EtOH	Ethanol
EtOAc	Ethyl acetate
MeOH	Methanol
CHCl_3_	Chloroform
MDCK	Madin–Darby kidney cell model
PB	Plasma–protein barrier
